# Supplementary material for: Associations between Early Surgery and Postoperative Outcomes in Elderly Patients with Distal Femur Fracture: A Retrospective Cohort Study
Source: J Clin Med. 2021 Dec 11;10(24):5800. doi: 10.3390/jcm10245800 (PMC8705557; doi:10.3390/jcm10245800)
Supplement: Supplementary file 1 [file jcm-10-05800-s001.zip › jcm-1415502-supplementary.pdf]

**Supplemental Table S1.** International Classification of Diseases Tenth Revision (ICD-10) codes for complications

| Complications | ICD-10 codes |
|---|---|
| Surgical site infection | T793, T814 |
| Acute coronary syndrome | I20–I25 |
| Heart failure | I50 |
| Respiratory disorder | J12–J18, J95–J96 |
| Sepsis | A40–A41, D65 |
| Pulmonary embolism | I26 |
| Deep venous thrombosis | I801–I802 |
| Cerebrovascular disease | I60–I64 |
| Renal failure | N17–N19 |
| Urinary tract infection | N390 |
| Disseminated intravascular coagulation | D65 |
| Pressure ulcer | L89 |

**Supplemental Table S2.** International Classification of Diseases Tenth Revision (ICD-10) codes for comorbidities

| Comorbidities | ICD-10 codes |
|---|---|
| Charlson comorbidity index | |
| Myocardial infarction | I21–I22, I252 |
| Chronic heart failure | I43, I50, I099, I110, I130, I132, I255, I420, I425–I429, P290 |
| Peripheral vascular disease | I70–I71, I731, I738–I739, I771, I790, I792, K551, K558–K559, Z958–Z959 |
| Cerebrovascular disease | I6, G45–G46, H340 |
| Dementia | F00–F03, F051, G30, G311 |
| Chronic pulmonary disease | J4, J60–J67, I278–I279, J684, J701, J703 |
| Rheumatic disease | M05–M06, M32–M34, M315, M351, M353, M360 |
| Peptic ulcer | K25–K28 |
| Mild liver dysfunction | B18, K73–K74, K700–K703, K709, K713–K715, K717, K760, K762–K764, K768–K769, Z944 |
| Diabetes mellitus without complications | E100–E101, E106, E108–E111, E116, E118–E121, E126, E128–E131, E136, E138–E141, E146, E148–E149 |
| Diabetes mellitus with complications | E102–E105, E107, E112–E115, E117, E122–E125, E127, E132–E135, E137, E142–E145, E147 |
| Hemiplegia | G81–G82, G041, G114, G801–G802, G830–G834, G839 |
| Renal dysfunction | N18–N19, I120, I131, N032-N037, N052–N057, N250, Z490–Z492, Z940, Z992 |
| Malignancy | C0–C3, C5–C6, C9, C40–C41, C43, C45–C49, C70–C76, C81–C85, C88 |
| Severe liver dysfunction | I85, I864, I982, K704, K711, K721, K729, K765–K767 |
| Metastasis | C77–C80 |
| Human immunodeficiency virus | B20–B22, B24 |

**Supplemental Table S3.** Postoperative complications in the original unmatched cohort and matched cohort

| Complications | Unmatched cohort | | Matched cohort | | |
|---|---|---|---|---|---|
| | Early surgery (≤2 days) *n* = 1,384 | Delayed surgery (≥3 days) *n* = 8,294 | Early surgery (≤2 days) *n* = 1,382 | Delayed surgery (≥3 days) *n* = 1,382 | *p*-value |
| Surgical site infection, *n* (%) | 10 (0.7) | 157 (1.9) | 16 (1.2) | 16 (1.2) | >0.999 |
| Acute coronary syndrome, *n* (%) | 19 (1.4) | 122 (1.5) | 10 (0.7) | 26 (1.9) | 0.007 |
| Heart failure, *n* (%) | 33 (2.4) | 176 (2.1) | 18 (1.3) | 19 (1.4) | 0.870 |
| Respiratory disorder, *n* (%) | 6 (0.4) | 40 (0.5) | 33 (2.4) | 27 (2.0) | 0.430 |
| Sepsis, *n* (%) | 6 (0.4) | 31 (0.4) | 6 (0.4) | 6 (0.4) | 1.000 |
| Pulmonary embolism, n (%) | 53 (3.8) | 406 (4.9) | 6 (0.4) | 4 (0.3) | 0.530 |
| Deep venous thrombosis, *n* (%) | 6 (0.4) | 55 (0.7) | 53 (3.8) | 72 (5.2) | 0.082 |
| Cerebrovascular disease, *n* (%) | 5 (0.4) | 24 (0.3) | 6 (0.4) | 7 (0.5) | 0.780 |
| Renal failure, *n* (%) | 25 (1.8) | 228 (2.7) | 5 (0.4) | 2 (0.1) | 0.260 |
| Urinary tract infection, *n* (%) | 2 (0.1) | 11 (0.1) | 25 (1.8) | 40 (2.9) | 0.060 |
| Disseminated intravascular coagulation, *n* (%) | 25 (1.8) | 211 (2.5) | 2 (0.1) | 1 (0.1) | 0.560 |
| Pressure ulcer, *n* (%) | 16 (1.2) | 91 (1.1) | 25 (1.8) | 39 (2.8) | 0.077 |

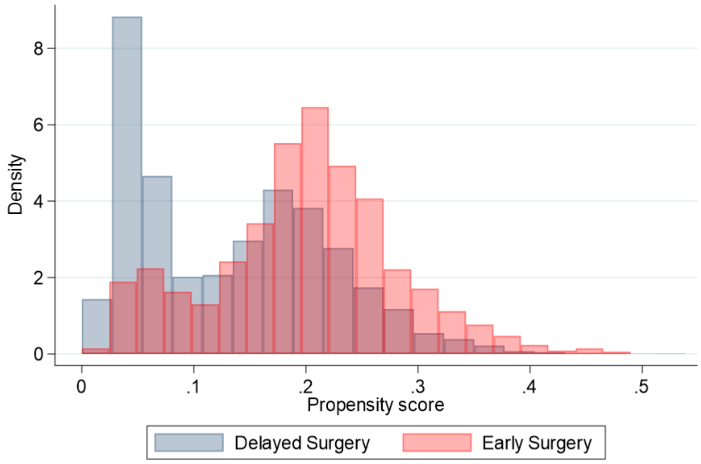


**Figure S1**. Distribution of propensity scores before matching.

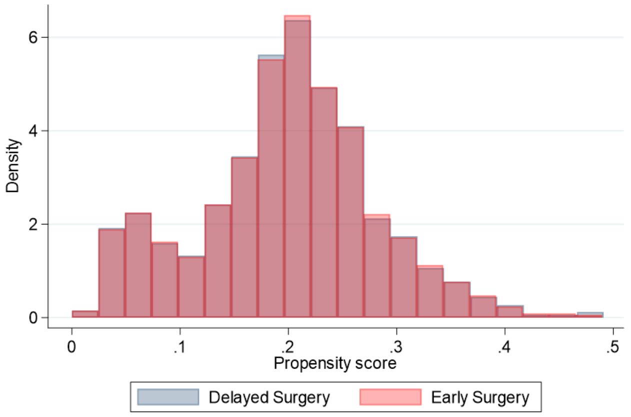


**Figure S2.** Distribution of propensity scores after matching.

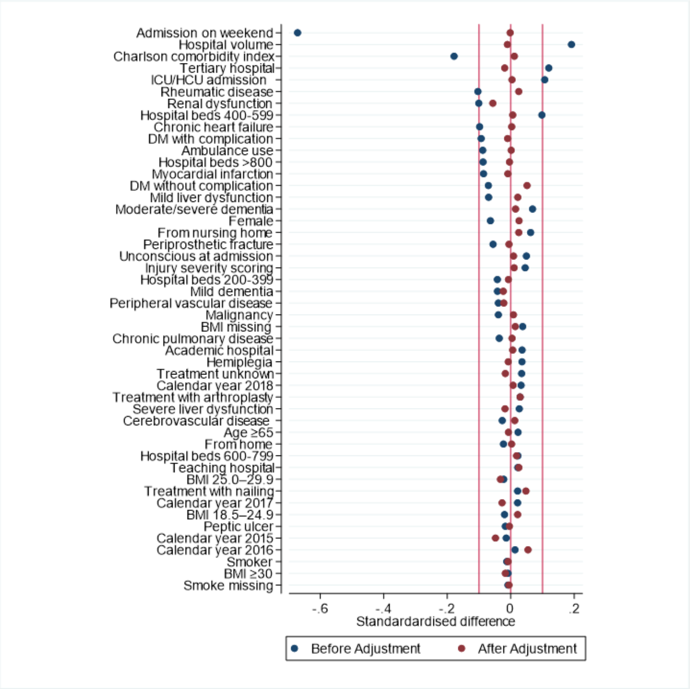


**Figure S3.** Kissing plot in propensity score matching.
